# Disseminated multiloculated hydatid cyst: a rare presentation

**DOI:** 10.11604/pamj.2023.44.197.39294

**Published:** 2023-04-24

**Authors:** Mrinmayee Vijay Mayekar, Neha Phate

**Affiliations:** 1 Department of Respiratory Medicine, Datta Meghe Institute of Higher Education and Research, Wardha, Maharashtra, India

**Keywords:** Multiloculated, hydatid cyst, dissemination

## Image in medicine

An 84-year-old male came to the respiratory Outpatient Department (OPD) with complaints of breathlessness of Modified Medical Research Council (MMRC) grade I-II for 6 months, swelling of approximately 7 x 6 x 5 cm over the lateral aspect of the chest wall, hoarseness of voice for 1 month, blackish discoloration of right foot for 1 month and chest pain on the right side for 10 days. He was a chronic smoker and farmer by occupation. On examination, he was conscious, oriented and vitally stable. His routine laboratory parameters were within normal range. He underwent high-resolution computed tomography (HRCT) thorax and abdomen which was suggestive of multiple well-defined hypodense lesions with mildly enhancing wall and internal septa noted in the left lung, liver, left adrenal gland, spleen, peritoneum with chest wall extension. Emphasizing on the age, we were suspecting malignancy which turned out to be disseminated hydatid cyst involving multiple organs and also extending to the chest wall on the left side which is pretty much less in occurrence. He started on tablet Albendazole and planned for video-assisted thoracoscopic surgery for mediastinal hydatid cyst. Disseminated hydatid cyst involving chest wall is pretty rare and account for only 2% of cases as per literature suggests.

**Figure 1:**
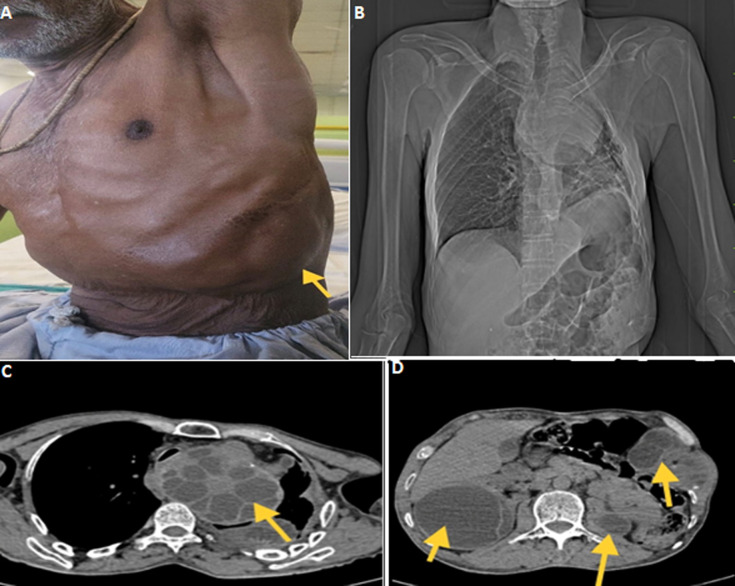
A) chest wall swelling of approximately 8 x 6 cm on the left side; B) scanogram of chest showing left paracardiac superior and inferior mediastinal radio-opaque circular lesion; C,D) multiple well-defined hypodense lesions with mildly enhancing wall and internal septa noted in the left lung, liver, left adrenal gland, spleen, peritoneum with chest wall extension

